# Supplementary material for: DeepPhospho accelerates DIA phosphoproteome profiling through in silico library generation
Source: Nat Commun. 2021 Nov 18;12:6685. doi: 10.1038/s41467-021-26979-1 (PMC8602247; doi:10.1038/s41467-021-26979-1)
Supplement: Supplementary file 1 — Supplementary Information [file 41467_2021_26979_MOESM1_ESM.pdf]

Supplementary Information for Manuscript

DeepPhospho accelerates DIA phosphoproteome profiling through *in silico* library generation

Ronghui Lou[1,2,3‡], Weizhen Liu[4‡], Rongjie Li[4], Shanshan Li[1], Xuming He[4,5*], Wenqing Shui[1,2*]

[1]iHuman Institute, ShanghaiTech University, Shanghai 201210, China

[2]School of Life Science and Technology, ShanghaiTech University, Shanghai 201210, China

[3]University of Chinese Academy of Sciences, Beijing 100049, China

[4]School of Information Science and Technology, ShanghaiTech University, Shanghai 201210, China

[5]Shanghai Engineering Research Center of Intelligent Vision and Imaging, Shanghai 201210, China

[‡]Equal contribution

*To whom correspondence should be addressed to:

Wenqing Shui Email: shuiwq@shanghaitech.edu.cn

Xuming He Email: hexm@shanghaitech.edu.cn

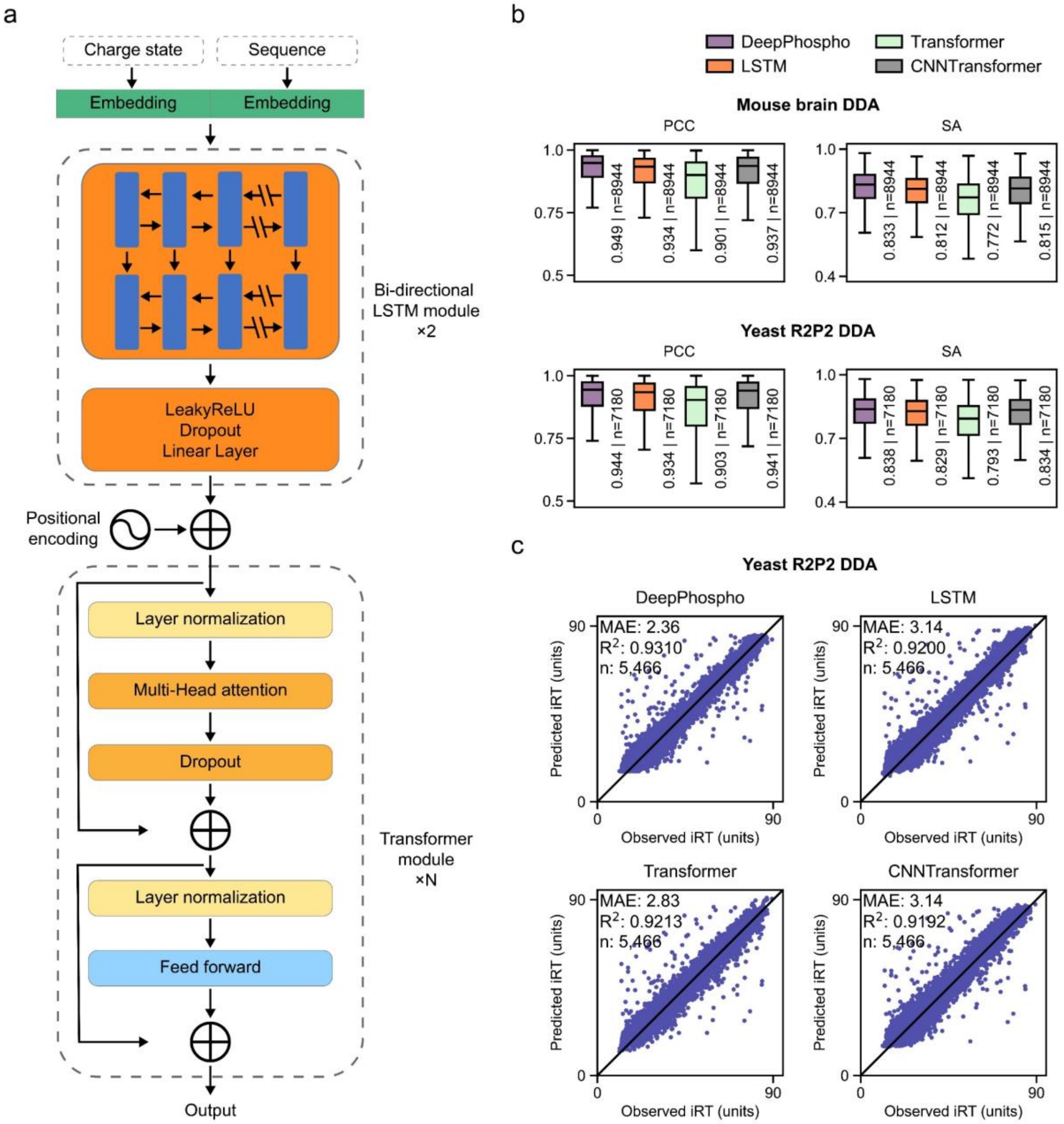

Supplementary Figure 1 Architecture of DeepPhospho and comparison with other baselines in the ablative study.

(a) Detailed architecture of DeepPhospho. For fragment ion intensity and iRT prediction, the embedded features first pass through two stacked bi-directional LSTMs, each of which is followed by a LeakyReLU-Dropout-Linear Layer. After the position encoding is added, the output of biLSTM module is fed into the Transformer module. The first part of each Transformer module is a layer-normalization layer, which is followed by the Multi-Head attention to capture global patterns and a dropout layer to prevent the overfitting. The Transformer module also adopts two skip connections to allow effective model training.

(b) Evaluation of DeepPhospho and three other baselines based on the distribution of Pearson correlation coefficient (PCC) and spectral contrast angle (SA) calculated between predicted and experimental MSMS spectra from mouse brain DDA and yeast R2P2 DDA datasets. Median PCC and SA are displayed; n is the number of phosphopeptides in the test set. Boxplot center line, median; box limits, upper and lower quartiles; whiskers, 1.5 × interquartile range. **(c)** Evaluation of DeepPhospho and three other baselines based on the correlation of predicted and experimental iRT values from the yeast R2P2 DDA data. Correlation coefficient of linear regression (R^2) and median absolute error (MAE) are displayed. Source data are provided as a Source Data file.

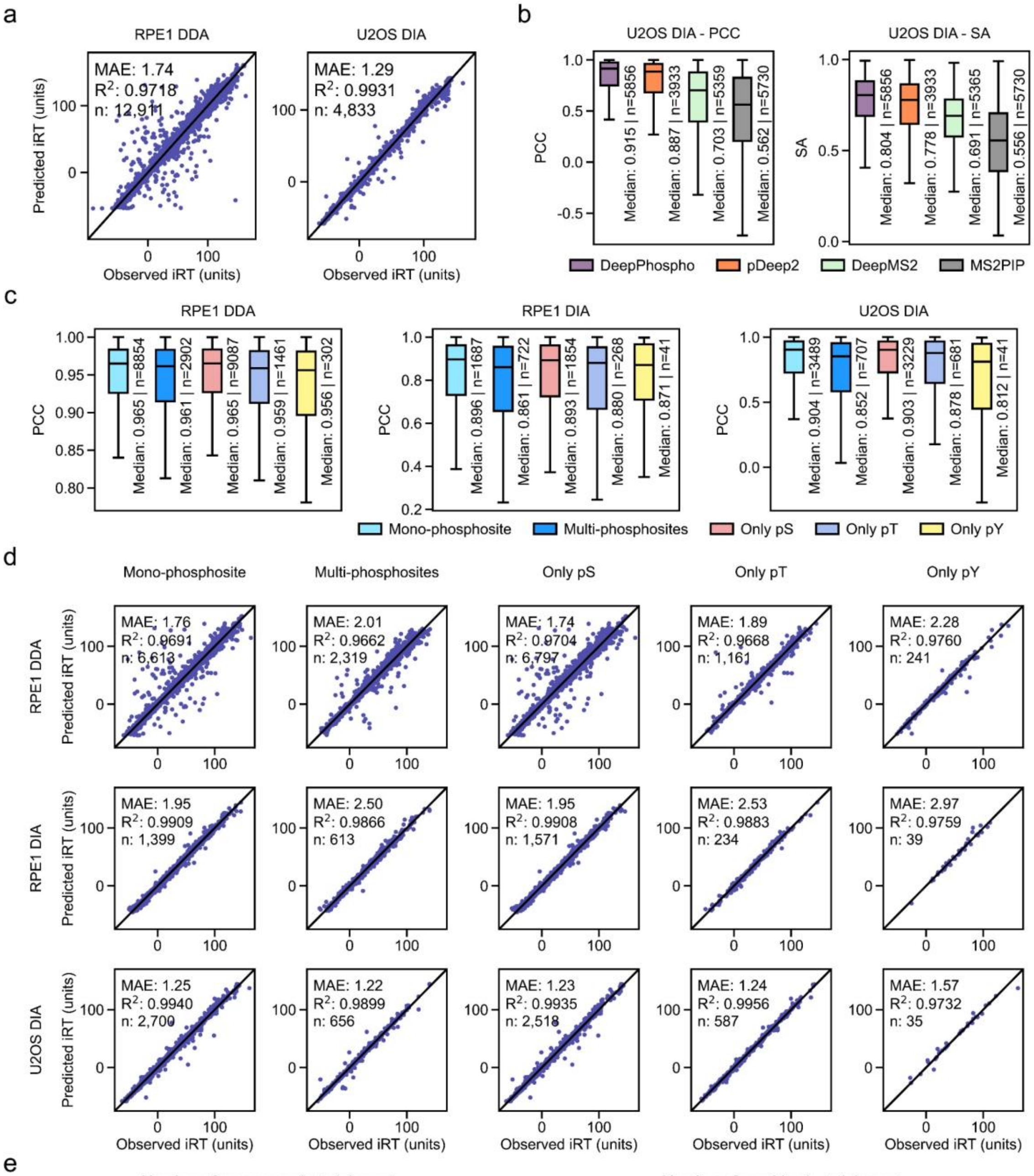

e

Number of precursors in training set

	Mono-phosphosite	Multi-phosphosites	Only pS	Only pT	Only pY
RPE1 DDA	70,805	23,260	72,648	11,904	2,331
RPE1 DIA	13,569	5,848	15,102	2,217	296
U2OS DIA	27,682	6,034	25,527	5,780	336

Number of peptides in training set

	Mono-phosphosite	Multi-phosphosites	Only pS	Only pT	Only pY
RPE1 DDA	53,310	18,247	54,425	9,407	1,854
RPE1 DIA	11,195	4,978	12,399	1,926	249
U2OS DIA	21,977	4,940	20,236	4,653	299

Supplementary Figure 2 Evaluation of DeepPhospho with other datasets and for different categories of phosphopeptides.

(a) Evaluation of DeepPhospho based on the correlation of predicted and experimental iRT values from RPE1 DDA and U2OS DIA datasets. R2 and MAE are indicated. **(b)**

Evaluation of DeepPhospho and three other models based on the distribution of PCC and SA calculated between predicted and experimental MSMS spectra from the U2OS DIA data. Median PCC and SA are indicated; **(c, d)** Evaluation of DeepPhospho predictions of fragment ion intensity (c) and iRT (d) for mono- or multi-phosphosite peptides and for phosphopeptides merely containing pS, pT or pY. Model performance was evaluated with RPE1 DDA, RPE1 DIA and U2OS DIA data. (e) Number of precursors used for training the fragment ion intensity model (left) and number of phosphopeptides used for training the iRT model (right). Phosphopeptides in different categories are separately analyzed. Boxplots: center line, median; box limits, upper and lower quartiles; whiskers, 1.5 × interquartile range. n is the number of phosphopeptides in the test set. Source data are provided as a Source Data file.

a

Peptide index	Synthetic phosphopeptide sequence	Precursor charge	PCC	
			Pred-Syn	Pred-Lib
1	S(ph)SSESYTQSFQSR	2	0.923	-0.614
2	NYGS(ph)PLISGSTPK	2	0.93	-0.394
3	AAS(ph)SAAQGAFQGN	2	0.89	-0.044
4	RVS(ph)PLNLSSVTP	2	0.824	-0.037
5	S(ph)LQQLAEER	2	0.969	0.117
6	S(ph)VGGSGGGSFGDNLVTR	2	0.786	0.148
7	NSFLGS(ph)PR	2	0.877	0.199

b

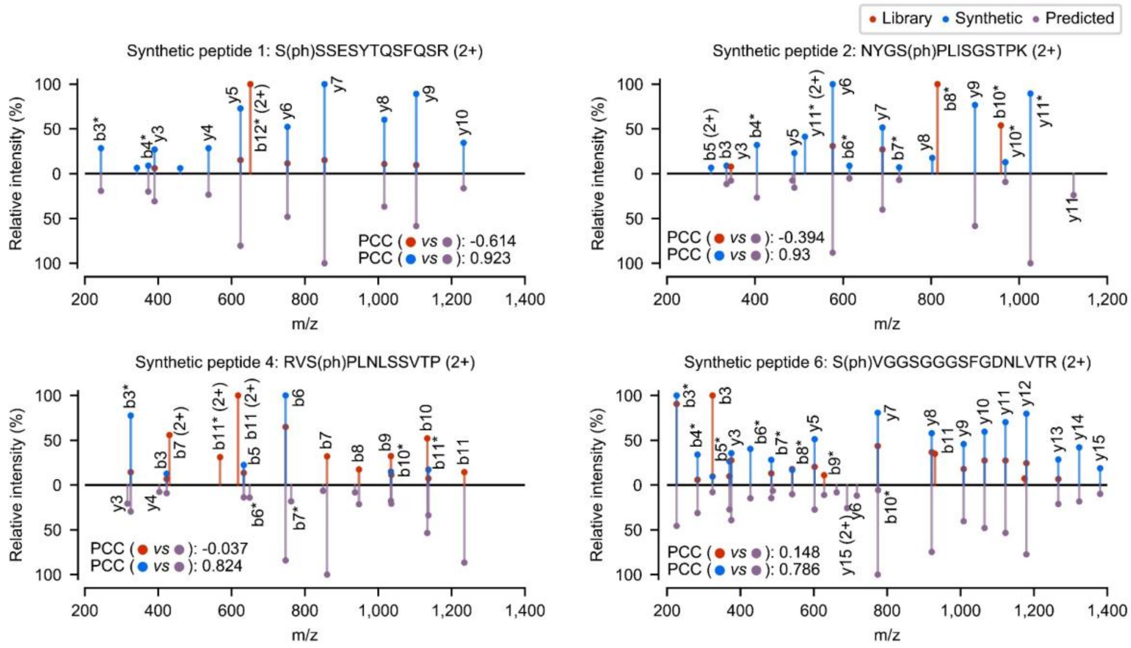

Supplementary Figure 3 Spectral similarity analysis for seven selected phosphopeptides.

(a) Sequences, charge states and PCC analysis of seven phosphopeptides. Correlation is calculated between the predicted spectra and the high-quality spectra of the synthetic peptide (Pred-Syn), and between the predicted spectra and the DIA library spectra (Pred-Lib). **(b)** Spectra mirror plots for four phosphopeptides not shown in Fig. 2C. Relative fragment ion intensities in the predicted spectra, the DIA library spectra and the synthetic peptide spectra are annotated by purple, orange and blue lines. * indicates the loss of a phosphate. Source data are provided as a Source Data file.

Condition index	Peptide length	Precursor m/z	Fragment m/z	Precursor charge	Max site number
1	7-59	unlimited	unlimited	2/3/4	3
2	7-26	350-1650	200-1800	2/3	1
3	7-26	350-1650	200-1800	2/3	2
4	7-26	350-1650	200-1800	2/3/4	1
5	7-26	350-1650	200-1800	2/3/4	2
6	7-26	350-1650	200-1800	2/3/4	3
7	7-26	436-1100	200-1800	2/3	1
8	7-26	436-1100	200-1800	2/3	2
9	7-26	436-1100	200-1800	2/3/4	1
10	7-26	436-1100	200-1800	2/3/4	2
11	7-52	350-1650	200-1800	MostFreq	3
12	7-52	350-1650	200-1800	2/3	1
13	7-52	350-1650	200-1800	2/3	2
14	7-52	350-1650	200-1800	2/3	3
15	7-52	350-1650	200-1800	2/3/4	3
16	7-52	350-1650	330-1381	2/3/4	3
17	7-52	436-1100	200-1800	2/3	2
18	7-52	436-1100	200-1800	2/3/4	1
19	7-52	436-1100	200-1800	2/3/4	2
20	7-52	436-1100	200-1800	2/3/4	3
21	7-52	436-1100	330-1381	2/3/4	3

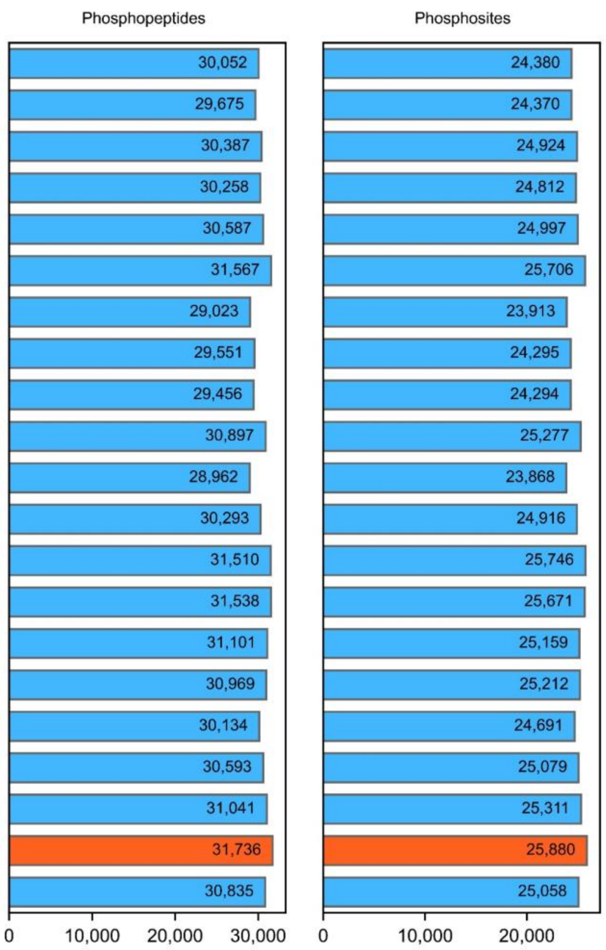

Supplementary Figure 4 Testing 21 different conditions in generating the predicted library hPhosPepDB contained in Lib 4 for U2OS DIA data analysis.

Left table summarizes all 21 combinations of peptide length, precursor and fragment m/z ranges, precursor charge and max phosphosite number for the library generation. Right column graphs show the total number of identified phosphopeptides and phosphosites from the U2OS DIA data with each predicted library generated under a specific condition. Condition 20 was selected as the best one for generation of Lib 4 used for U2OS DIA data analysis. The max site number (1, 2 or 3) indicates the max number of phosphosites present in all peptides in the library. A max site number of 1 indicates only mono-site phosphopetides are included in the library while a max site number of 3 indicates peptides with 1-3 phosphosites are all included.

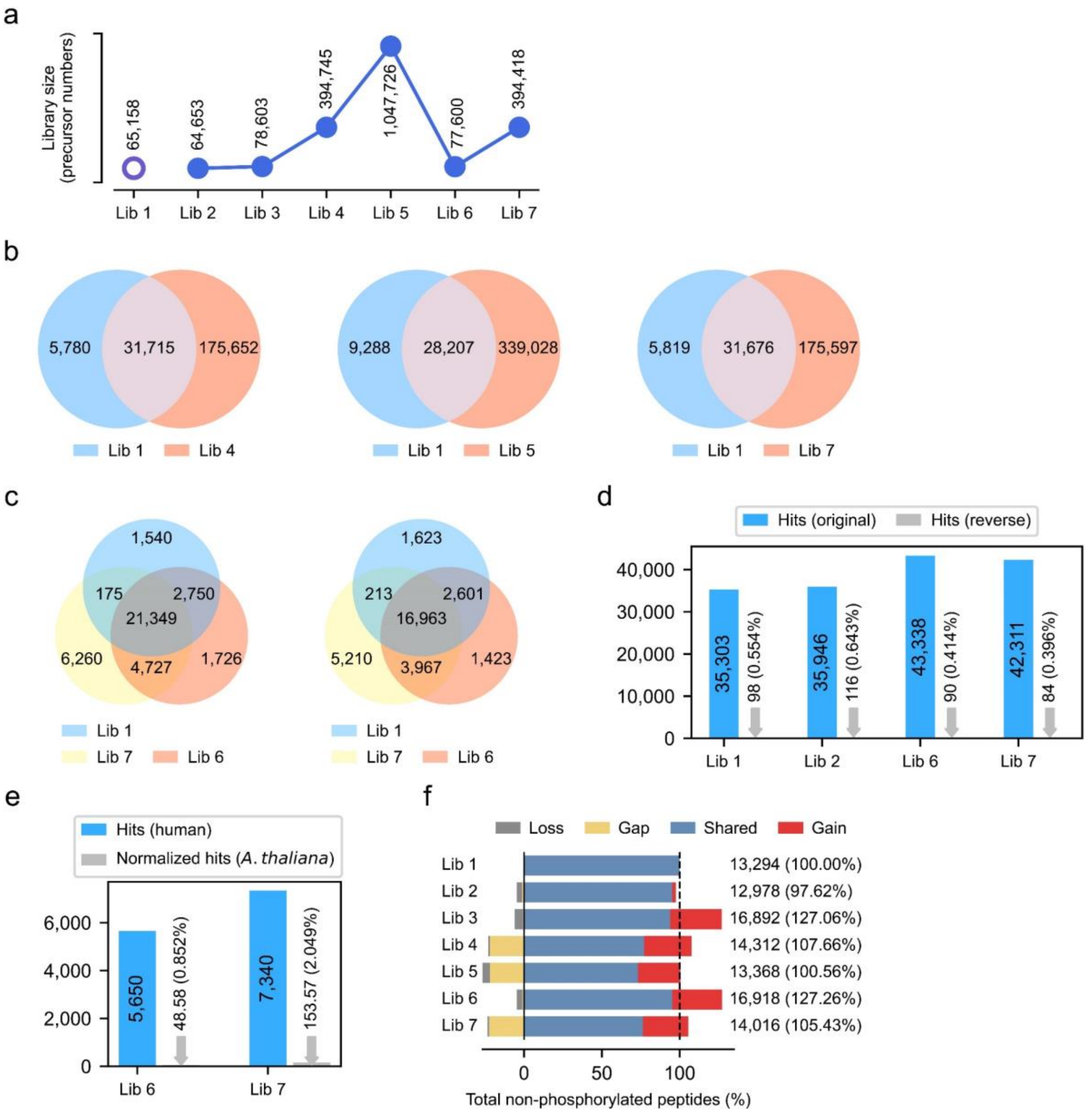

Supplementary Figure 5 Comparison of spectral libraries and phosphoproteome profiling results from U2OS DIA data analysis.

(a) Number of total peptide precursors in each generated library. **(b)** Overlapping and unique phosphopeptides present in a DeepPhospho predicted library (Lib 4, Lib 5, Lib 7) *vs* Lib 1. **(c)** Overlapping and unique phosphopeptides (left) or phosphosites (right) identified from U2OS DIA data with Lib 6 and Lib 7 *vs* Lib 1. **(d)** Library-specific FDR assessed using an original-reverse combined library. Number of peptide IDs in the U2OS DIA data analysis is shown for the original or the reverse sub-library, with the calculated FDR indicated as a percentage. **(e)** FDR assessed with a two-species library. Number of

peptide IDs is shown for the predicted human phosphoproteome sub-library or the predicted *A. thaliana* phosphoproteome sub-library, with the calculated FDR indicated as a percentage. **(f)** Number of non-phosphorylated peptides identified from the U2OS DIA data analysis with each library. Percentage of the total non-phosphorylated peptides number is shown for each predicted library relative to Lib 1. The proportions of shared identifications (IDs), gained IDs, lost IDs and gap IDs yielded by Lib 2 to Lib 7 compared to Lib 1 are indicated in different color. Gap IDs are those present in Lib1 yet absent in the DeepPhospho predicted libraries, thus they cannot be identified with the latter. Source data are provided as a Source Data file.

Condition index	Peptide length	Precursor m/z	Fragment m/z	Precursor charge	Max site number
1	7-59	unlimited	unlimited	2/3/4	3
2	7-27	472-1145	200-2000	2/3	1
3	7-27	472-1145	200-2000	2/3	2
4	7-27	472-1145	200-2000	2/3/4	1
5	7-27	472-1145	200-2000	2/3/4	2
6	7-27	472-1145	200-2000	2/3/4	3
7	7-27	501-1068	200-2000	2/3	1
8	7-27	501-1068	200-2000	2/3	2
9	7-27	501-1068	200-2000	2/3/4	1
10	7-27	501-1068	200-2000	2/3/4	2
11	7-52	472-1145	200-2000	MostFreq	3
12	7-52	472-1145	200-2000	2/3	1
13	7-52	472-1145	200-2000	2/3	2
14	7-52	472-1145	200-2000	2/3	3
15	7-52	472-1145	200-2000	2/3/4	3
16	7-52	472-1145	333-1325	2/3/4	3
17	7-52	501-1068	200-2000	2/3	2
18	7-52	501-1068	200-2000	2/3/4	1
19	7-52	501-1068	200-2000	2/3/4	2
20	7-52	501-1068	200-2000	2/3/4	3
21	7-52	501-1068	333-1325	2/3/4	3

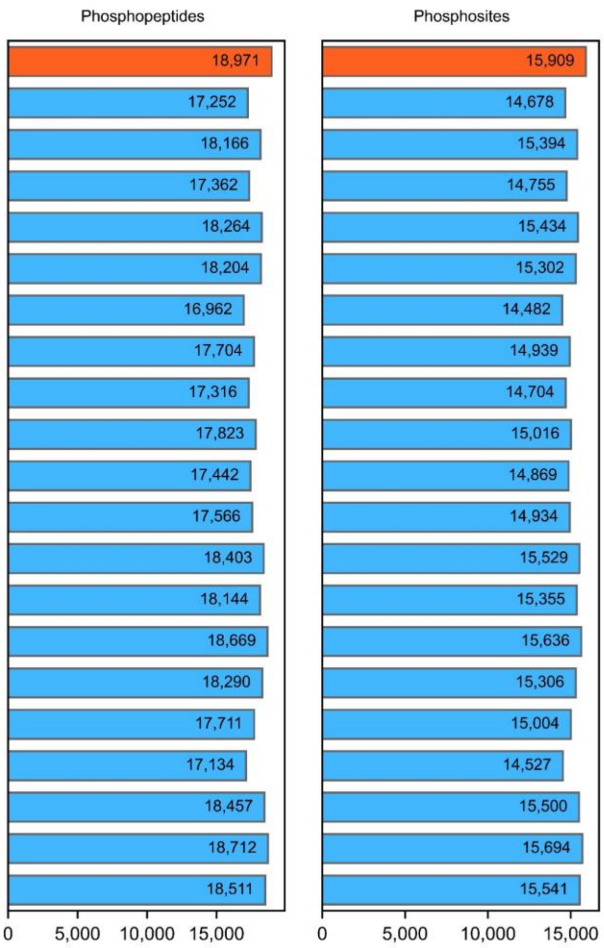

Supplementary Figure 6 Testing 21 different conditions in generating the predicted library hPhosPepDB contained in Lib 4 for RPE1 DIA data analysis.

Left table summarizes all 21 combinations of peptide length, precursor and fragment m/z ranges, precursor charge and max phosphosite number for the library generation. Right column graphs show the total number of identified phosphopeptides and phosphosites from the U2OS DIA data with each predicted library generated under a specific condition. Condition 1 was selected as the best one for generation of Lib 4 used for RPE1 DIA data analysis.

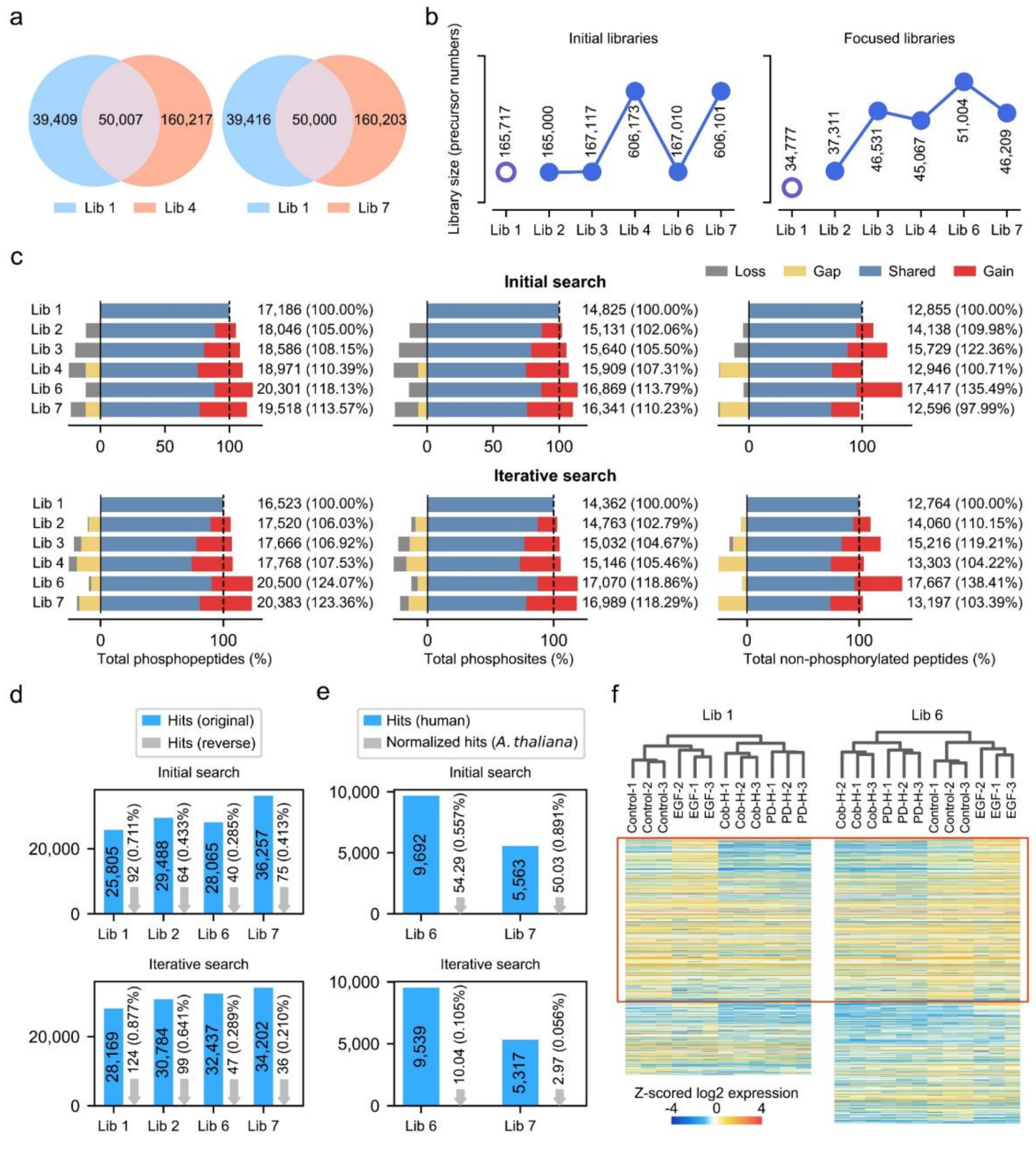

Supplementary Figure 7 Comparison of spectral libraries and phosphoproteome profiling results from RPE1 DIA data analysis.

(a) Overlapping and unique phosphopeptides identified from RPE1 DIA data with Lib 6 or Lib 7 *vs* Lib 1. **(b)** Number of total peptide precursors in each initial library and the corresponding focused library. **(c)** Number of total phosphopeptides (left), total phosphosites (middle), and total non-phosphorylated peptides (right) identified from RPE1 DIA data with each library in the initial search (upper panel) or in the iterative search (lower panel). Percentage of the total number of identifications is shown for each predicted library

relative to Lib 1. The proportions of shared IDs, gained IDs, lost IDs and gap IDs yielded by Lib 2 to Lib 7 compared to Lib 1 are indicated in different color. **(d)** Library-specific FDR assessed using an original-reverse combined library. Number of peptide IDs in the RPE1 DIA data analysis is shown for the original or the reverse sub-library, with the calculated FDR indicated as a percentage. **(e)** FDR assessed with a two-species library. Number of peptide IDs is shown for the predicted human phosphoproteome sub-library or the predicted *A. thaliana* phosphoproteome sub-library, with the calculated FDR indicated as a percentage. **(f)** Unsupervised hierarchical clustering of significantly regulated phosphosites yielded at different stimulation conditions with Lib 1 or Lib 6. The red rectangle indicates phosphosites co-identified by two libraries. Source data are provided as a Source Data file.

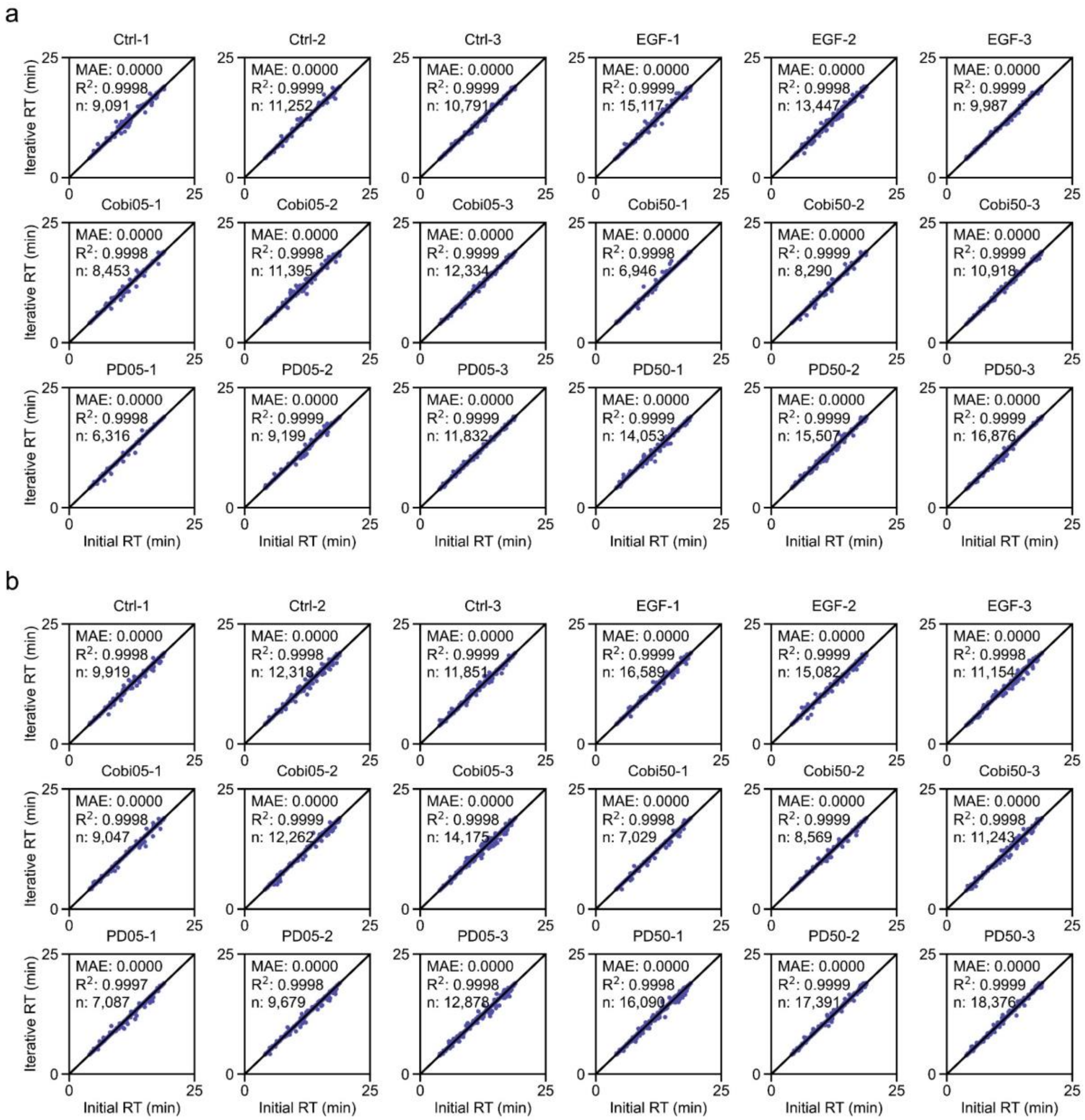

Supplementary Figure 8 RT correlation of co-identified peptides in the initial and it erative searches of RPE1 DIA data. RT correlation is shown for peptides identified with Lib 1 **(a)** or Lib 7 **(b)** in each DIA run of the dataset. n is the number of peptides in the test set. Source data are provided as a Source Data file.

a

	True phosphopeptides	True phosphosites	TRR (%)	False phosphosites	FLR (%)
SynLib	154	164	93.18	0	0
predSynLib	154	164	93.18	0	0
SynLib+RPE1 DDA (initial)	136	141	80.11	5	3.42
SynLib+RPE1 DDA (iterative)	154	164	93.18	3	1.8
predSynLib+hPhosPepDB (initial)	130	133	75.57	10	6.99
predSynLib+hPhosPepDB (iterative)	156	166	94.32	5	2.92
predSynLib+hPhosSiteDB (initial)	121	124	70.45	9	6.77
predSynLib+hPhosSiteDB (iterative)	156	166	94.32	6	3.49

TRR (True recovery rate) = N(true phosphosites) / 176 (number of total known phosphosites)

FLR (False localization rate) = N(false phosphosites) / (N(true phosphosites) + N(false phosphosites))

b

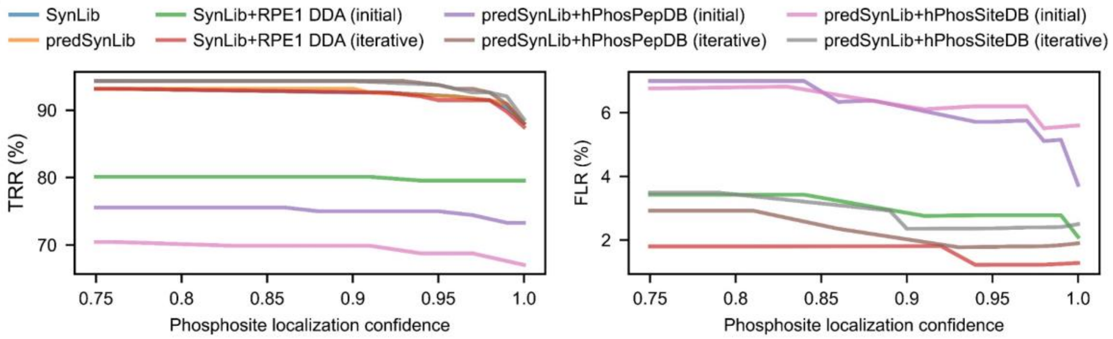

c

	True phosphopeptides	True phosphosites	False phosphosites	FLR (%)
SynLib	253	260	0	0
predSynLib	241	248	0	0
SynLib+Yeast DDA (initial)	153	157	4	2.48
SynLib+Yeast DDA (iterative)	257	265	2	0.75
predSynLib+yPhosPepDB (initial)	179	184	12	6.12
predSynLib+yPhosPepDB (iterative)	252	261	8	2.97

d

Human synthetic phosphopeptide dataset

	False phosphopeptides	False phosphosites
hPhosSiteDB	186	169
hPhosPepDB	223	136
RPE1 DDA	149	61

Yeast synthetic phosphopeptide dataset

	False phosphopeptides	False phosphosites
yPhosPepDB	903	508
Yeast DDA	418	237

Supplementary Figure 9 FLR estimation using synthetic phosphopeptide DIA data sets.

(a) Summary of true and false phosphosites identified with each library and the calculated TRR and FLR for a human phosphopeptide dataset. SynLib, an experimental DDA library comprised of 166 synthetic phosphopeptides containing 176 known phosphosites; predSynLib, a predicted library built on the synthetic phosphopeptide information in SynLib;

SynLib+RPE1 DDA, a hybrid experimental library combing SynLib with an extensive human phosphoproteome library RPE1 DDA; predSynLib+hPhosPepDB and predSynLib+hPhosSiteDB, hybrid predicted libraries combining predSynLib and a large predicted library built on a public database. Results are shown for the initial search with SynLib or predSynLib and initial/iterative searches with a hybrid library, all at a phosphosite localization confidence >0.75. **(b)** TRR and FLR as a function of the phosphosite localization confidence cut-off for DIA data analysis with each library listed in (a). **(c)** Summary of true and false phosphosites identified with each library and calculated FLR for a yeast phosphopeptide dataset. SynLib, an experimental DDA library comprised of 300 synthetic phosphopeptides containing 321 known phosphosites; predSynLib, a predicted library built on the synthetic phosphopeptide information in SynLib; SynLib+Yeast DDA, a hybrid experimental library combing SynLib with an extensive yeast phosphoproteome DDA library; predSynLib+yPhosPepDB, hybrid predicted libraries combining predSynLib and a predicted library built on a public database. **(d)** Number of false phosphopeptides and false phosphosites present in different libraries used to analyze the human phosphopeptide dataset (left) or the yeast phosphopeptide dataset (right).

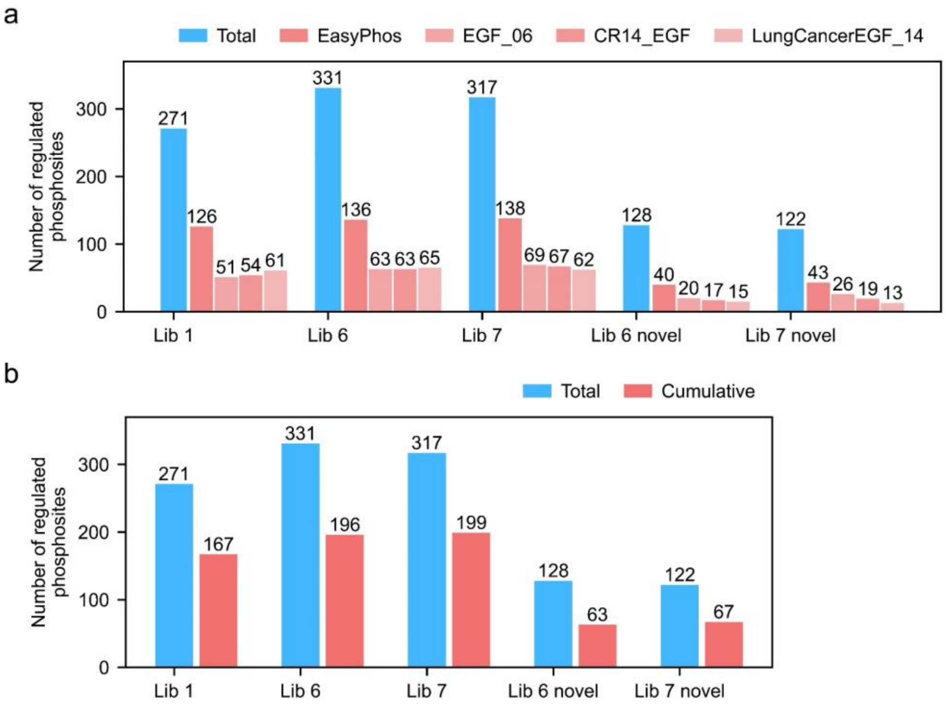

Supplementary Figure 10 Comparison of regulated phosphosites reported in this study (blue bars) and in four published EGF signaling proteomics studies (red bars).

(a) Number of total regulated phosphosites and those also reported in each previous study (EasyPhos[1], EFG_06[2], CR14_EGF[3], LungCancerEGF_14[4]) revealed with the DDA library (Lib 1) or two predicted libraries (Lib 6 and Lib 7). Novel regulated phosphosites revealed by Lib or Lib 7 and reported in the previous study are also shown. **(b)** The cumulative number of regulated phosphosites reported in previous studies (red) and number of total regulated phosphosites revealed with each library (blue). Notice that the cumulative novel EGF-regulated phosphosites that were repeatedly found in previous studies are 63 and 67, nearly or above half of the total novel phosphosites revealed by Lib 6 and Lib 7. Moreover, data mining with the two predicted libraries uncovered more regulated phosphosites than Lib 1 (331 and 317 *vs* 271) with a percentage of verifiable sites very similar to Lib 1.

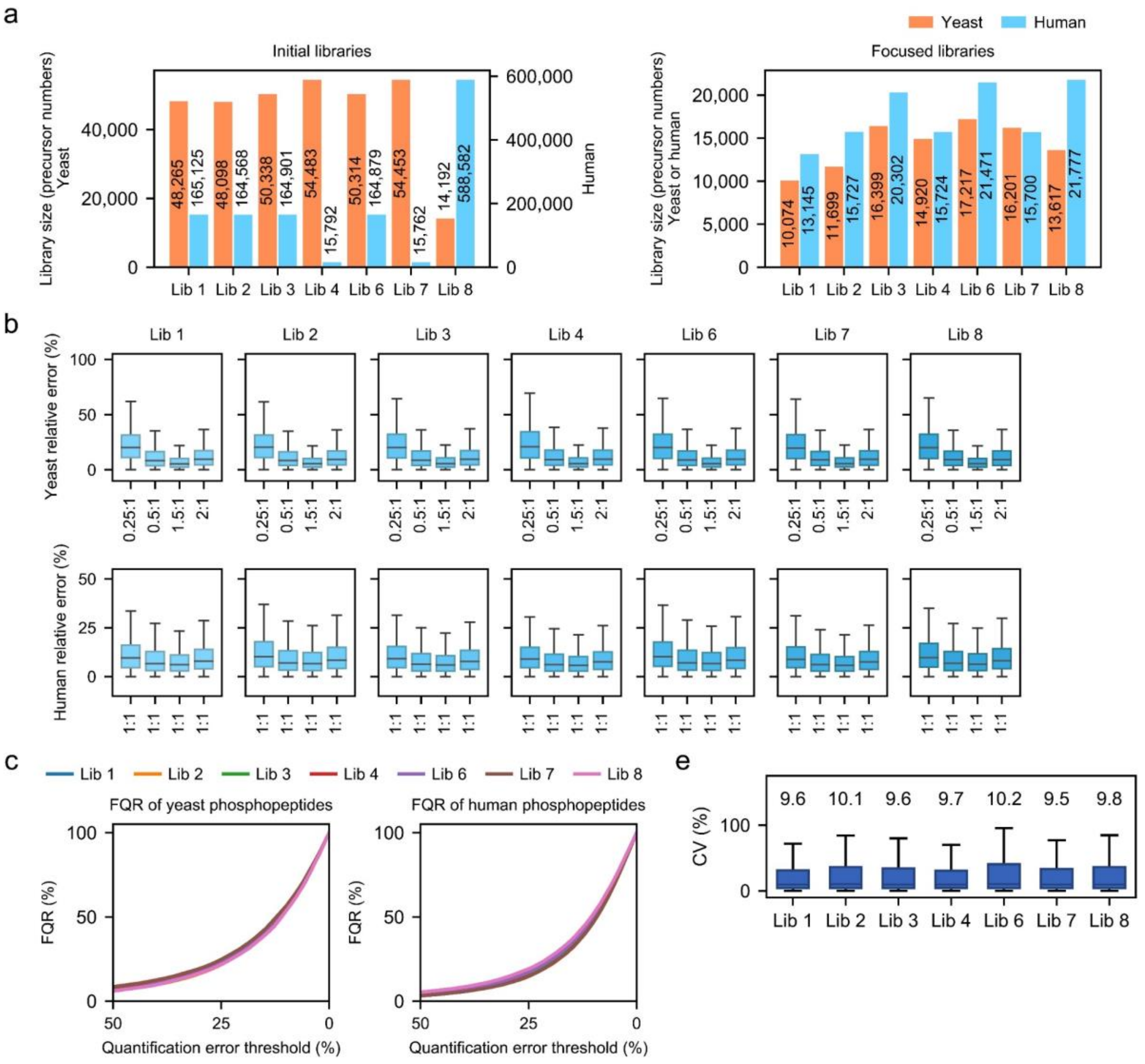

d

Yeast	Lib 1	Lib 2	Lib 3	Lib 4	Lib 6	Lib 7	Lib 8
50% quant error threshold	6.2	6.2	6.6	8.5	7.1	8.2	6.1
30% quant error threshold	16.6	16.4	16.6	19.4	17.7	19.3	16.5

Human	Lib 1	Lib 2	Lib 3	Lib 4	Lib 6	Lib 7	Lib 8
50% quant error threshold	3.7	4.2	3.8	3.5	4.2	3.2	5.3
30% quant error threshold	11.4	12.4	11.4	10.6	12.7	10.5	14.3

Supplementary Figure 11 Comparison of spectral libraries and phosphoproteome quantification results from DIA data analysis of the two-proteome model.

(a) Number of yeast and human peptide precursors in each initial library and the corresponding focused library. **(b)** Boxplots of relative errors between measured and expected ratios for yeast peptides (upper) and human peptides (lower) from search results with each library. Ratios were calculated based on the mean quantities in 6 replicates of each sample. **(c)** FQR as a function of the quantification error threshold for yeast

phosphopeptides (left) and human phosphopeptides (right) identified with different libraries. **(d)** FQR percentages at a 50% or 30% quantification error threshold for yeast phosphopeptides (upper) and human phosphopeptides (lower) identified with different libraries. **(e)** Coefficient of variation (CV) of all phosphopeptide quantification with different libraries between 6 replicates at each dilution condition. Median CV% is indicated above the box plot. In b and e, boxplot center line, median; box limits, upper and lower quartiles; whiskers, 1.5 × interquartile range. Source data are provided as a Source Data file.

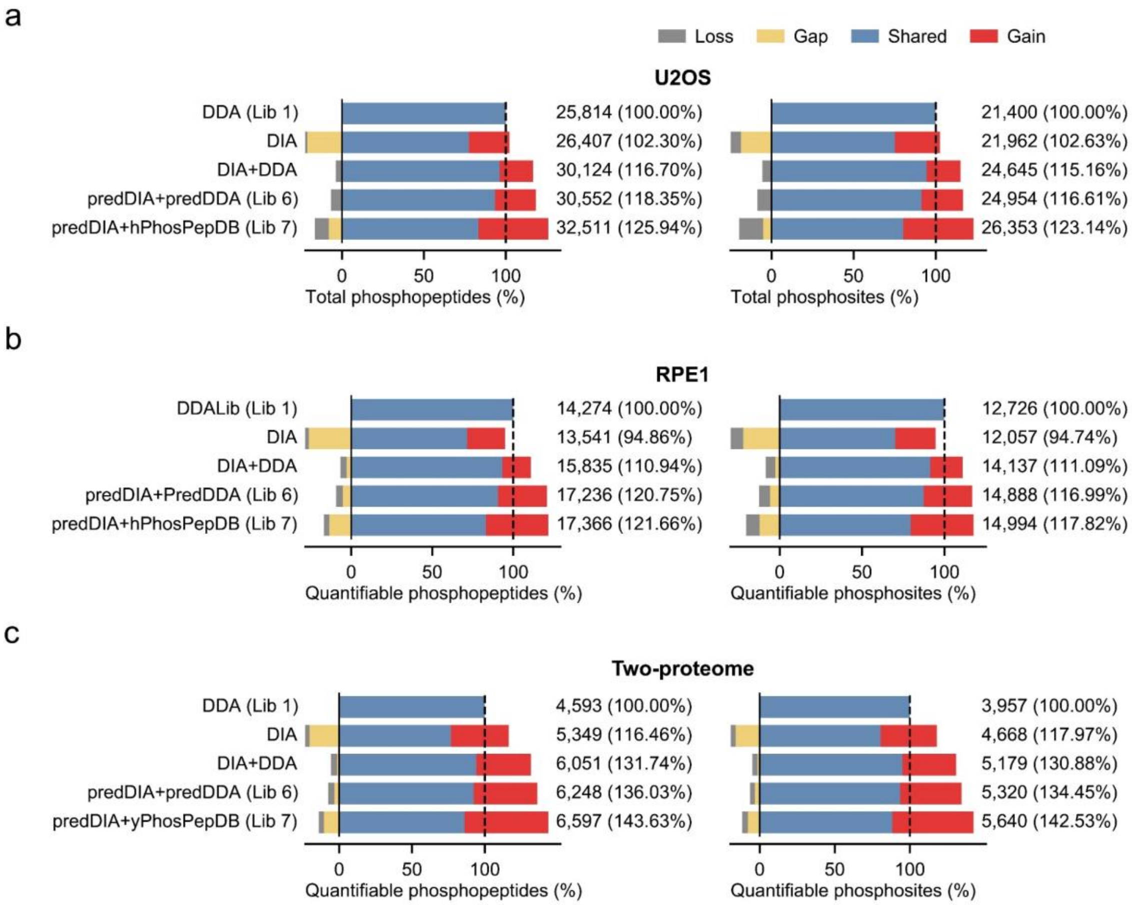

Supplementary Figure 12 Number of phosphopeptides and phosphosites identified using two other experimental libraries in comparison to Lib 1, Lib 6 and Lib 7. DIA and DIA+DDA library refer to the direct DIA library and the merged DIA and DDA library respectively, both built on the experimentally acquired DIA or DDA MS data. The initial search result is shown for the U2OS data while the iterative search results are shown for the RPE1 and two-proteome model data. The proportions of shared identifications (IDs), gained IDs, lost IDs and gap IDs yielded by different libraries compared to Lib 1 are indicated in different color.

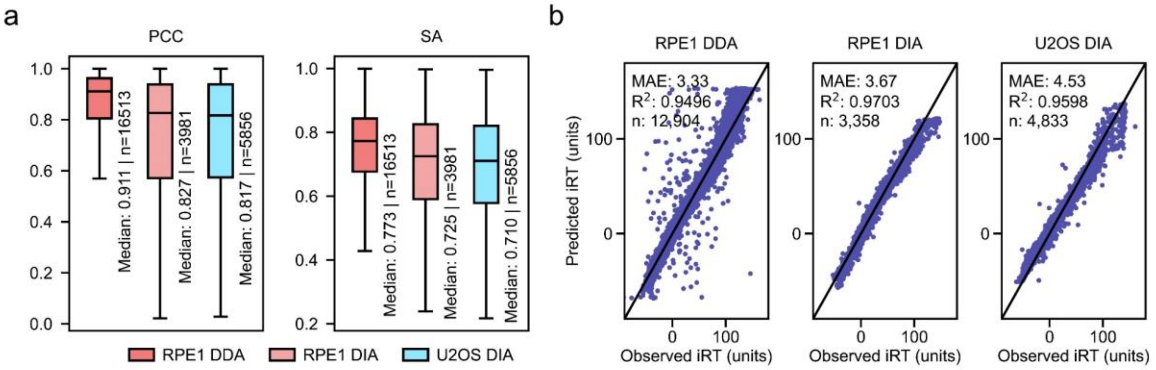

c

	Number of parameters (M)
DeepPhospho (LSTM+Transformer)	37.84
pDeep2	7.04
DeepMS2	0.21
DeepPhospho RT Ensemble	168.16
LSTM (ablation study)	21.02
Transformer (ablation study)	6.33
CNNTransformer (ablation study)	46.29

d

Number of precursors used for ion intensity model pre-training

	Precursors for training	Precursors for test
Mouse brain DDA data	80,494	8,945
VeroE6 DIA data	48,602	5,402
Yeast DIA data	38,980	4,332

Number of peptides used for retention time model pre-training

	Peptides for training	Peptides for test
Human phosphopeptide RT data	184,102	20,456
Mouse brain DDA data	64,172	3,378
VeroE6 DIA data	39,064	4,341
Yeast DIA data	32,227	3,581

Supplementary Figure 13 Evaluation of DeepPhospho pre-trained models and model information.

(a) Evaluation of the pre-trained fragment ion intensity model based on PCC (left) and SA (right) analysis with three test sets. Boxplot center line, median; box limits, upper and lower quartiles; whiskers, 1.5 × interquartile range. **(b)** Evaluation of the pre-trained iRT model based on iRT correlation analysis with three test sets. To deal with chromatography variation in different data sets, we randomly selected ten peptide-iRT pairs at five iRT percentiles (10%, 30%, 50%, 70%, 90%) and calibrated the predicted iRTs by second-order polynomial fitting. **(c)** Total number of model parameters in DeepPhospho, pDeep2, DeepMS2 and three models assessed in the ablation study. **(d)** Number of precursors and

peptides used for DeepPhospho pre-training.

n is the number of phosphopeptides in the test set. Source data are provided as a Source Data file.

Supplementary References

1. Humphrey, S.J., Karayel, O., James, D.E. & Mann, M. High-throughput and high-sensitivity phosphoproteomics with the EasyPhos platform. *Nat Protoc* **13**, 1897-1916 (2018).
2. Olsen, J.V. et al. Global, in vivo, and site-specific phosphorylation dynamics in signaling networks. *Cell* **127**, 635-648 (2006).
3. Sharma, K. et al. Ultradeep human phosphoproteome reveals a distinct regulatory nature of Tyr and Ser/Thr-based signaling. *Cell Rep* **8**, 1583-1594 (2014).
4. Zhang, X. et al. Identifying novel targets of oncogenic EGF receptor signaling in lung cancer through global phosphoproteomics. *Proteomics* **15**, 340-355 (2015).
