# Supplementary material for: DeepPhospho accelerates DIA phosphoproteome profiling through in silico library generation
Source: Nat Commun. 2021 Nov 18;12:6685. doi: 10.1038/s41467-021-26979-1 (PMC8602247; doi:10.1038/s41467-021-26979-1)
Supplement: Supplementary file 3 — Description of Additional Supplementary Files [file 41467_2021_26979_MOESM3_ESM.docx]

File Name: Supplementary Data 1

Description: Overview of MS datasets and phosphoproteome databases

File Name: Supplementary Data 2

Description: Three phosphoproteome databases built in this study

File Name: Supplementary Data 3

Description: EGF-regulated sites identified in RPE1 cells using different libraries
