# Supplementary material for: DeepPhospho accelerates DIA phosphoproteome profiling through in silico library generation
Source: Nat Commun. 2021 Nov 18;12:6685. doi: 10.1038/s41467-021-26979-1 (PMC8602247; doi:10.1038/s41467-021-26979-1)
Supplement: Supplementary file 7 — Reporting Summary [file 41467_2021_26979_MOESM7_ESM.pdf]

nature research

Corresponding author(s): Xuming He, Wenqing Shui

Last updated by author(s): Oct 24, 2021

Reporting Summary

Nature Research wishes to improve the reproducibility of the work that we publish. This form provides structure for consistency and transparency in reporting. For further information on Nature Research policies, see our Editorial Policies and the Editorial Policy Checklist.

Statistics

For all statistical analyses, confirm that the following items are present in the figure legend, table legend, main text, or Methods section.

n/a	Confirmed	
☐	☒	The exact sample size (*n*) for each experimental group/condition, given as a discrete number and unit of measurement
☐	☒	A statement on whether measurements were taken from distinct samples or whether the same sample was measured repeatedly
☐	☒	The statistical test(s) used AND whether they are one- or two-sided<br>*Only common tests should be described solely by name; describe more complex techniques in the Methods section.*
☒	☐	A description of all covariates tested
☐	☒	A description of any assumptions or corrections, such as tests of normality and adjustment for multiple comparisons
☐	☒	A full description of the statistical parameters including central tendency (e.g. means) or other basic estimates (e.g. regression coefficient) AND variation (e.g. standard deviation) or associated estimates of uncertainty (e.g. confidence intervals)
☒	☐	For null hypothesis testing, the test statistic (e.g. *F*, *t*, *r*) with confidence intervals, effect sizes, degrees of freedom and *P* value noted<br>*Give P values as exact values whenever suitable.*
☒	☐	For Bayesian analysis, information on the choice of priors and Markov chain Monte Carlo settings
☐	☒	For hierarchical and complex designs, identification of the appropriate level for tests and full reporting of outcomes
☐	☒	Estimates of effect sizes (e.g. Cohen's *d*, Pearson's *r*), indicating how they were calculated

Our web collection on statistics for biologists contains articles on many of the points above.

Software and code

Policy information about availability of computer code

Data collection	Public MS raw data were downloaded from PRIDE via FileZilla (3.51.0). The raw data were processed either with MaxQuant (1.6.14.0), MaxQuant (1.6.17.0), Spectronaut (14.5), Skyline (20.2.0.343), or directly used the result files provided on PRIDE (the downloaded RPE1 DDA library and two-proteome DDA library were initially built with Spectronaut v11.0.15038.19 and v13.0.190309, respectively).
Data analysis	DeepPhospho model was implemented in Python (3.7.9) with PyTorch (1.7.1), numpy (1.19.2), scipy (1.6.0), and pandas (1.2.1). The source code of DeepPhospho is available at https://github.com/weizhenFrank/DeepPhospho.<br>Other models used in this work are: pDeep2 (https://github.com/pFindStudio/pDeep/tree/master/pDeep2, accessed Mar 2019), DeepMS2 (https://github.com/lmsac/DeepDIA, accessed Dec 2020) with DeepMS2-phospho supported (https://github.com/lmsac/DeepMS2-phospho, accessed Dec 2020), MS2PIP (server: https://iomics.ugent.be/ms2pip, accessed Dec 2020).<br>Data analysis were performed with python, numpy, scipy, and pandas as those used for implementing DeepPhospho, and statsmodels (0.12.0) was also used. In addition, PerseusR was used to perform Peptide Collapse (https://github.com/AlexHgO/Perseus_Plugin_Peptide_Collapse). Visualization were based on matplotlib (3.3.2), matplotlib-venn (0.11.5), and seaborn (0.10.1).

For manuscripts utilizing custom algorithms or software that are central to the research but not yet described in published literature, software must be made available to editors and reviewers. We strongly encourage code deposition in a community repository (e.g. GitHub). See the Nature Research guidelines for submitting code & software for further information.

Data

Policy information about availability of data

All manuscripts must include a data availability statement. This statement should provide the following information, where applicable:

- Accession codes, unique identifiers, or web links for publicly available datasets
- A list of figures that have associated raw data
- A description of any restrictions on data availability

Raw DDA and PRM data from synthetic phosphopeptide analysis, DeepPhospho generated spectral libraries, and DIA search results have been deposited to the ProteomeXchange Consortium via the iProX partner repository with the dataset identifier IPX0003513000 [https://www.iprox.cn/page/project.html?id=IPX0003513000] and equivalent to PXD028601 [http://proteomecentral.proteomexchange.org/cgi/GetDataset?ID=PXD028601]. Source data are provided with this paper.

Public MS data used in this work are as followings: PXD006637 [https://www.ebi.ac.uk/pride/archive/projects/PXD006637] (mouse brain DDA), PXD019113 [https://www.ebi.ac.uk/pride/archive/projects/PXD019113] (Vero E6 DIA), PXD013453 [https://www.ebi.ac.uk/pride/archive/projects/PXD013453] (yeast R2P2), PXD014525 [https://www.ebi.ac.uk/pride/archive/projects/PXD014525] (RPE1, two-proteome), PXD017476 [https://www.ebi.ac.uk/pride/archive/projects/PXD017476] (U2OS), PXD009227 [https://www.ebi.ac.uk/pride/archive/projects/PXD009227] (U-87 DDA), PXD019797 [https://repository.jpostdb.org/entry/JPST000859] (human synthetic phosphopeptide dataset), PXD004573 [https://www.ebi.ac.uk/pride/archive/projects/PXD004573] (yeast synthetic phosphopeptide dataset). All MS raw data were downloaded from PRIDE FTP site via FileZilla (v3.51.0) or from jPOST via Mozilla Firefox.

Databases used in this work are: UniProt [https://www.uniprot.org], EPSD [http://epsd.biocuckoo.cn], PhosphoSitePlus [https://www.phosphosite.org/staticDownloads], PhosPhAt [http://phosphat.uni-hohenheim.de], Reactome [https://reactome.org].

Field-specific reporting

Please select the one below that is the best fit for your research. If you are not sure, read the appropriate sections before making your selection.

[x] Life sciences   [ ] Behavioural & social sciences   [ ] Ecological, evolutionary & environmental sciences

For a reference copy of the document with all sections, see nature.com/documents/nr-reporting-summary-flat.pdf

Life sciences study design

All studies must disclose on these points even when the disclosure is negative.

Sample size	Samples analyzed in this work are synthetic phosphopeptides, and the peptide mixture was injected for either DDA or PRM MS data acquisition. All published datasets analyzed in this work contained the full sample sizes which are specified in Figures 3, 4 and 6. No sample size calculation is performed. All used raw data are listed in Supplementary Table 1.
Data exclusions	Raw data of some public MS datasets were selected from the entire datasets to ensure they are suitable for specific usages, and the finally used raw data files were listed in Supplementary Table 1. The processed data from MaxQuant, Spectronaut, and Skyline were passed the general criteria of data filtration.
Replication	Attempts to test DeepPhospho performance by analyzing all replications in three datasets are successful: the U2OS DIA data has 2 conditions with 10 replicates at each condition; the RPE1 DIA data has 6 conditions with 3 replicates at each condition; the two-proteome model data has 5 dilution ratios with 6 replicates at each condition.
Randomization	All datasets used for model training were randomly split to a training set, a validation set and a test set at a ratio of 8:1:1.
Blinding	Not applicable. This study focuses on the establishment of a new strategy for DIA-MS based phosphoproteome data mining.

Reporting for specific materials, systems and methods

We require information from authors about some types of materials, experimental systems and methods used in many studies. Here, indicate whether each material, system or method listed is relevant to your study. If you are not sure if a list item applies to your research, read the appropriate section before selecting a response.

Materials & experimental systems

n/a	Involved in the study
[x]	[ ] Antibodies
[x]	[ ] Eukaryotic cell lines
[x]	[ ] Palaeontology and archaeology
[x]	[ ] Animals and other organisms
[x]	[ ] Human research participants
[x]	[ ] Clinical data
[x]	[ ] Dual use research of concern

Methods

n/a	Involved in the study
[x]	[ ] ChIP-seq
[x]	[ ] Flow cytometry
[x]	[ ] MRI-based neuroimaging
